# Spread of dual-class drug-resistant *Mycoplasma genitalium* in Tokyo, Japan, 2023–2025

**DOI:** 10.1128/aac.01367-25

**Published:** 2025-12-30

**Authors:** Ryuha Omachi, Kazuo Imai, Akihiro Sato, Masashi Tanaka, Hitomi Mizushina, Keita Takeuchi, Nobuaki Mori, Takuya Maeda

**Affiliations:** 1 Department of Clinical Laboratory Medicine, Saitama Medical University 73443, Saitama, Japan; 2 Department of Infectious Disease and Infection Control, Saitama Medical University 13031, Saitama, Japan; 3 KARADA Internal Medicine Clinic, Tokyo, Japan; 4 Department of Medicine, Division of Clinical Infectious Diseases, Showa Medical University School of Medicine 13059 , Tokyo, Japan; Columbia University Irving Medical Center, New York, New York, USA

**Keywords:** drug resistance, *Mycoplasma genitalium*, macrolide, quinolone

## Abstract

The increasing prevalence of *Mycoplasma genitalium* (MG) strains harboring macrolide and quinolone resistance-associated mutations (MRMs and QRMs, respectively) is a growing global concern. However, data on resistance patterns and genetic diversity in Japan remain limited. This study investigated MRMs and QRMs, genetic diversity using *mgpB* and *MG309* typing, and their association with treatment outcome in MG strains collected in Tokyo, Japan, between 2023 and 2025. Between 2023 and 2025, 188 clinical samples from 162 MG-positive patients were analyzed. Resistance mutations in 23S rRNA, *parC*, and *gyrA* were sequenced, and molecular typing was performed. Treatment outcomes were assessed using test-of-cure results. MRMs in 23S rRNA and QRMs in *parC* S83I and *gyrA* were identified in 94.4%, 65.5%, and 22.5% of samples, respectively. Dual-class resistance (MRMs + QRMs) was found in 89.4% of strains. Phylogenetic analysis based on *mgpB* and *MG309* typing revealed the emergence of dual-class drug-resistant clonal complexes, particularly those harboring *mgpB* alleles 79, 140, 161, and 184. Dual-QRMs were significantly associated with quinolone treatment failure (52.4% vs 23.5%, *P* = 0.016). Dual-class drug-resistant MG strains, including emerging clonal complexes, are spreading in Tokyo, Japan. These findings emphasize the need for continued molecular surveillance and prudent antimicrobial use to preserve treatment efficacy.

## INTRODUCTION

*Mycoplasma genitalium* (MG) is a sexually transmitted infection that can infect the urethra, cervix, and rectum (1). In Japan, urethral MG has been detected in 22% of men with symptomatic non-gonococcal urethritis (2). Under the Japanese national guidelines (3), MG testing is not performed routinely in all individuals undergoing screening for sexually transmitted infections. Testing is typically reserved for symptomatic patients with non-gonococcal urethritis who continue to exhibit symptoms despite treatment for *Chlamydia trachomatis*. Testing kits capable of detecting MG drug resistance genes are not yet widely available. Regarding treatment, the guidelines recommend first-line options of either a single 1 g dose of azithromycin or 200 mg/day doxycycline for 7 days. In cases of azithromycin resistance or treatment failure, 400 mg/day sitafloxacin for 7 days is recommended. If treatment fails, tetracycline may be administered for more than 14 days. Sequential treatment with tetracycline for 7 days followed by sitafloxacin for another 7 days is also suggested as an alternative regimen. Follow-up is generally based on the improvement of clinical symptoms. If a test of cure (TOC) is performed, it is recommended that it is conducted at 2 to 4 weeks after the completion of treatment for MG.

The increasing prevalence of macrolide and quinolone dual-class drug-resistant strains of MG has become a growing public health concern (4). Macrolide resistance-associated mutations (MRMs) of MG have been identified as single nucleotide mutations at positions A2058 and A2059 in the V region of the 23S rRNA gene (*Escherichia coli* numbering) (5). These MRMs induce structural changes in the ribosomal antibiotic binding site, thereby reducing the effectiveness of macrolide antibiotics (5). Quinolone resistance-associated mutations (QRMs) are linked to mutations in the quinolone resistance-determining regions of the *parC* and *gyrA* genes, which encode DNA topoisomerase IV and gyrase, respectively (6). The amino acid changes in the quinolone resistance-determining regions of *parC* and *gyrA* are associated with an increase in the minimum inhibitory concentration of moxifloxacin and sitafloxacin (6). Especially, amino acid changes in ParC (e.g., S83I) and GyrA (e.g., G93, M95, and D99) are associated with moxifloxacin and sitafloxacin treatment failure (7–9). Although high antimicrobial resistance rates of MG have been reported among the men who have sex with men (MSM) population in Tokyo (10, 11), data on resistance in the non-MSM population in Japan remain limited (12).

Evaluating the diversity of drug-resistant strains through molecular typing methods is essential for identifying high-risk populations, estimating transmission routes, and formulating effective intervention strategies. Sequence typing methods such as *mgpB* (MG191) adhesin gene single-nucleotide polymorphism typing (13) and *MG309* short tandem repeat typing (14) have shown high discriminatory power (15) and have been used widely for molecular surveillance globally (16–18). However, in Japan, the evaluation of MG strain diversity, including drug-resistant strains, using *mgpB* and/or *MG309* typing has not been conducted sufficiently to date (19, 20). To optimize the effectiveness of treatment and prevent the further spread of drug-resistant MG strains, it is important to monitor continuously the prevalence and diversity of resistance-associated mutations.

This study investigated the prevalence and diversity of MRMs and QRMs in MG as well as their association with treatment outcome in the clinical setting in Tokyo, Japan.

## MATERIALS AND METHODS

### Patients and sample collection

Adult patients (≥18 years old) diagnosed with MG infection at KARADA Internal Medicine Clinics (Shibuya and Gotanda, Tokyo, Japan) between 2023 and 2025 were enrolled in this study. These clinics provide insurance-covered and non-insurance-covered (out-of-pocket) medical services, including internal medicine and infectious disease care, such as for sexually transmitted infections. In 2024, the two clinics saw 24,686 and 41,210 patients, respectively. The male-to-female ratio was approximately 1:1. Approximately 3,000 and 7,000 *Neisseria gonorrhoeae* and *C. trachomatis* tests and 400 and 500 MG tests were performed annually at the two clinics, respectively. In this study, the following patients were included: (i) those with persistent symptoms despite testing negative for *N. gonorrhoeae* and *C. trachomatis*, (ii) those whose symptoms did not resolve after treatment for *N. gonorrhoeae* and *C. trachomatis*, and (iii) asymptomatic individuals who were known contacts of MG-positive partners. At the initial diagnosis, MG was detected by cobas MG/TV (Roche, Basel, Switzerland), multiplex PCR for STD Mycoplasma Nucleic Acid Testing (LSI Medience Corporation, Tokyo, Japan), and laboratory-developed quantitative PCR testing of the *MgPa* gene of MG (21). All TOCs were performed using the cobas MG/TV assay. Residual first-void urine and vaginal swab samples after routine testing were utilized in this study. The samples were stored at −80°C until DNA extraction. Patient information was collected retrospectively from the hospital electronic medical records. Data on risk of infection, such as the use of commercial sex workers, employment as a commercial sex worker, sexual activity with anonymous partners, and sexual activity with a regular partner, were obtained through a structured questionnaire completed by patients at their initial visit. The questionnaire included an option to decline to answer. This study was reviewed and approved by Saitama Medical University Hospital (approval number: 2023-026). Informed consent was obtained by opt-out via the hospital’s website.

### DNA extraction

First-void urine and suspension of vaginal swabs (1 mL) were centrifuged at 15,000 × *g* for 15 min at room temperature, and the sediment was resuspended in 140 μL phosphate-buffered saline. DNA was extracted from 140 μL of the suspension using a QIAamp Viral RNA Mini Kit (QIAGEN, Hilden, Germany), which is recommended by the manufacturer for DNA extraction from urine samples instead of a QIAamp DNA Mini Kit. The DNA was eluted in 60 μL of 10 mM Tris-HCl buffer (pH 8.5).

### Sequencing of drug resistance-associated genes and *mgpB-MG309* typing

23S rRNA, *gyrA*, *parC*, *mgpB*, and *MG309* were analyzed in this study. Nested PCR amplification of the genes (13, 14, 22, 23) was performed using KOD One PCR Master Mix (Toyobo, Tokyo, Japan). The primer sequences are shown in Table S1. The amplicons were analyzed by 1% agarose gel electrophoresis with ethidium bromide staining, and the PCR products were purified using an ExoSAP-IT (Applied Biosystems, Waltham, MA, USA) or QIAquick PCR Purification Kit (QIAGEN). Direct Sanger sequencing was performed by Eurofins Genomics (Tokyo, Japan).

### Sequencing analysis

The obtained sequence data of 23S rRNA, *gyrA*, and *parC* were compared with the corresponding regions of the MG reference strain G37 (GenBank: L43967.2) to identify mutations. The sequence types (STs) from *mgpB-MG309* typing were determined via the PubMLST BIGSdb database (https://pubmlst.org/organisms/mycoplasma-genitalium) (24). The phylogenetic tree of the concatenated *mgpB* and *MG309* sequence was constructed using IQtree ver. 2.4 (25). Phylogenetic trees were created using iTOL (https://itol.embl.de/).

### Statistical analysis

Continuous variables with a normal distribution are expressed as the mean (±standard deviation) and those with a non-normal distribution as the median (interquartile range) and were compared using Student’s *t*-test or the Mann–Whitney *U* test, as appropriate. Categorical variables are presented as frequency and percentage (%) and were compared using a chi-squared test or Fisher’s exact test, as appropriate. A two-sided *P*-value < 0.05 was considered statistically significant. All statistical analyses were conducted using R ver. 4.1.2 (The R Foundation for Statistical Computing, Vienna, Austria).

## RESULTS

### Baseline characteristics of the participants

During the study period, 162 patients with confirmed MG infections were enrolled, and a total of 188 clinical samples (152 first-void urine and 36 vaginal swabs) were collected. Multiple samples from the same patients were all obtained from the same anatomical sites at different time points during TOCs. The baseline characteristics of the study participants are shown in Table 1. Among the 162 patients, the median age was 30 years, with an interquartile range of 25–39 years, and 79.0% (*n* = 128) were male; 93.3% (*n* = 151) were symptomatic, whereas 6.7% (*n* = 11) were asymptomatic individuals who were known contacts of MG-positive partners. Regarding potential risk factors for infection, 33 patients (20.4%) reported engaging in sexual activity with commercial sex workers. Thirteen patients (8.0%) reported working as commercial sex workers. Sexual activity with anonymous partners was reported by 50 patients (30.9%), while 74 patients (45.7%) reported having sexual activity with a regular partner. The number of respondents who did not disclose information for each category was 29 (17.9%), 29 (17.9%), 51 (31.5%), and 51 (31.5%), respectively.

**TABLE 1:** Clinical characteristics of the study participants in 162 patients^*a*^

Characteristic	No. (%) or median [IQR]
Age, years, median [IQR]	30 [25–39]
Male, *n* (%)	128/162 (79.0)
Female, *n* (%)	34/162 (21.0)
Symptomatic, *n* (%)	151/162 (93.3)
Asymptomatic, *n* (%)	11/162 (6.7)
Risk of infection, *n* (%)	
Use of commercial sex workers	33/133* (24.8)
Working as a commercial sex worker	13/133* (9.8)
Sexual activity with anonymous partners	44/105** (41.9)
Sexual activity with a regular partner	74/111*** (66.7)
Initial treatment, *n* (%)	
Minocycline 7 days + sitafloxacin 7 days (sequential)	137/162 (84.6)
Moxifloxacin 10 days	5/162 (3.1)
Sitafloxacin 7 days	2/162 (1.2)
Minocycline 7 days	5/162 (3.1)
Doxycycline 7 days	2/162 (1.2)
Other	3/162 (1.9)
None	8/162 (4.9)
Initial treatment outcome, *n* (%)	
Cure	61/162 (37.7)
Failure	33/162 (20.4)
Unknown	68/162 (41.9)

^*a*^ Of the total, 29*, 57**, and 51*** patients had missing responses in the questionnaire. IQR, interquartile range.

The most common initial treatment regimen in 137 patients (84.6%) was the sequential administration of minocycline for 7 days followed by sitafloxacin for 7 days. Other regimens included moxifloxacin for 10 days (*n* = 5, 3.1%), sitafloxacin for 7 days (*n* = 2, 1.2%), minocycline for 7 days (*n* = 5, 3.1%), and doxycycline for 7 days (*n* = 2, 1.2%). Eight patients (4.9%) did not receive any initial treatment. Treatment outcome was assessed based on the results of a TOC conducted between 28 and 90 days following the initiation of treatment. Outcome data were available for 94 patients (58.0%), among whom 61 (64.9%) achieved microbiological cure, while 33 (35.1%) experienced treatment failure. The remaining 68 patients (42.0%) did not have TOC results available.

### Treatment outcome after initial treatment failure

**Fig 1:**
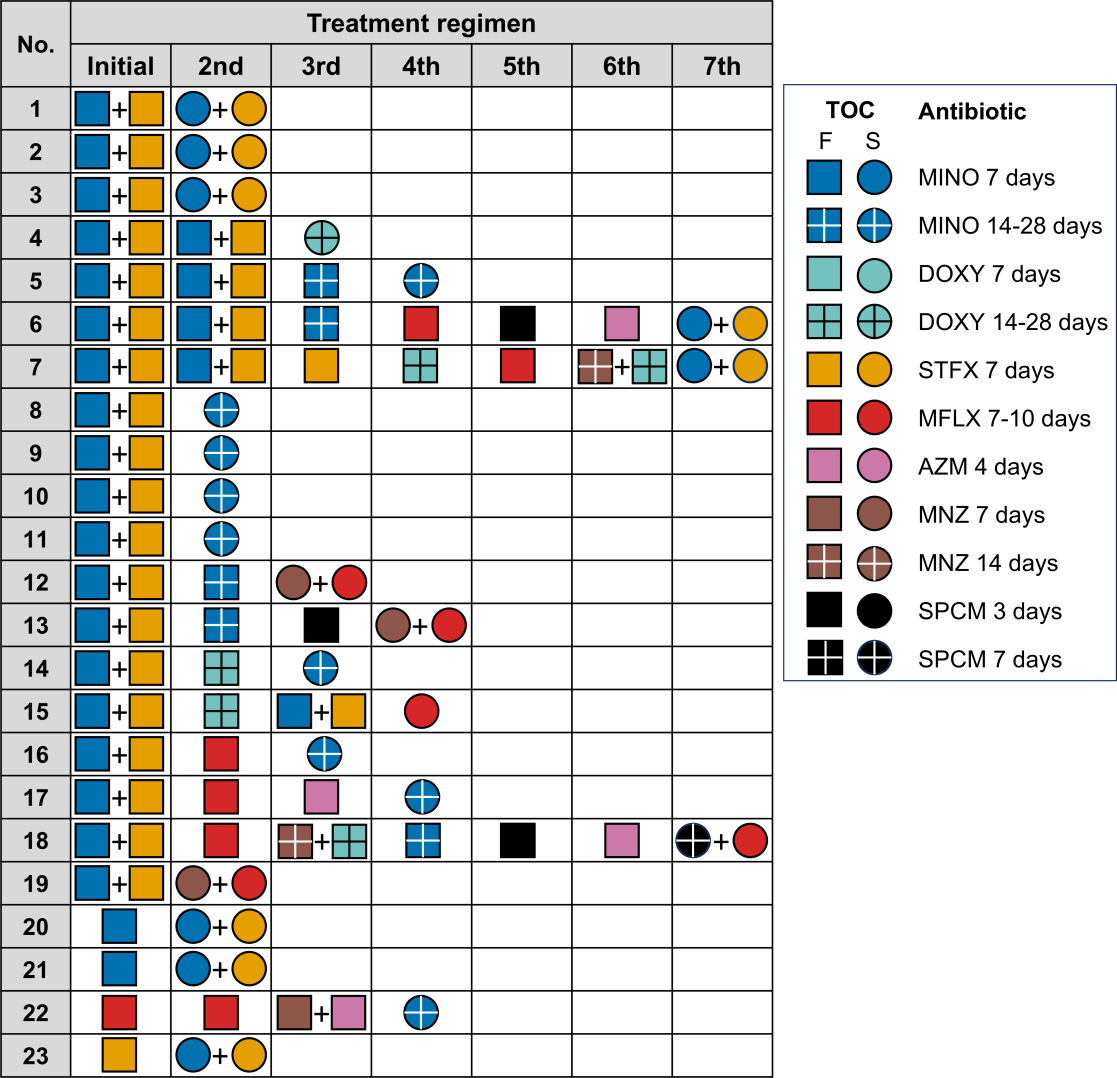
Treatment regimens and outcomes after initial treatment failure. Treatment regimens and microbiological outcomes of 23 patients who achieved microbiological cure following initial treatment failure. Each row represents a patient and lists the antibiotics administered at each treatment stage and the corresponding TOC result. The treatment regimens consisted of minocycline (MINO) and doxycycline (DOXY) (200 mg/day), moxifloxacin (MFLX) (400 mg/day), sitafloxacin (STFX) (200 mg/day), metronidazole (MNZ) (1 g/day), azithromycin (AZM) (1 g single dose, 500 mg/day for 3 days), and spectinomycin (SPCM) (4 g single dose, 2 g/day for 2 days [3-day regimen]; or 2 g/day for 7 days [7-day regimen]). In the TOC results, squares indicate failure (F) and circles indicate success (S).

Among the 33 patients who experienced initial treatment failure, 31 received additional treatment, and 23 of them finally achieved microbiological cure (Fig. 1). The number of treatment cycles required for cure ranged from 2 to 7. Of the 23 patients who were cured, 19 initially received the sequential administration of minocycline and sitafloxacin; the remaining 4 patients initially received monotherapy with either minocycline, moxifloxacin, or sitafloxacin. Among the 21 patients whose initial regimen included a fluoroquinolone, 11 were successfully treated with the re-administration of a fluoroquinolone-containing regimen. Specifically, six patients were cured with the sequential administration of minocycline and sitafloxacin. Three patients responded to a combination of moxifloxacin and metronidazole. One patient was cured with a combination of spectinomycin and moxifloxacin, and another with moxifloxacin monotherapy. The remaining 10 patients achieved cure through the long-term administration of either minocycline or doxycycline for 14 to 28 days. In contrast, the administration of azithromycin after the initial failure of a fluoroquinolone-containing regimen was unsuccessful in all cases.

### Analysis of resistance-associated genes

Of the 188 clinical samples, 23S rRNA, *parC*, and *gyrA* were successfully sequenced in 160 samples collected from 142 patients. The results of sequence analysis for the 142 clinical samples collected prior to initial treatment are summarized in Table 2. To avoid duplication, follow-up samples collected from the same patients at the time of a TOC were excluded. Among the 142 samples, mutations in 23S rRNA were highly prevalent (*n* = 134, 94.4%). The most common mutation was A2059G (*E. coli* numbering), found in 94 samples (66.2%), followed by A2058G in 29 samples (20.4%) and A2058T in 11 samples (7.7%). Wild-type (WT) sequences were observed in only eight samples (5.6%). Mutations in *parC* were also detected frequently in 133 samples (93.7%), particularly at codons 83 and 87. The S83I (G248T) mutation was the most common (*n* = 93, 65.5%). Other mutations included S83N (G248A, *n* = 20, 14.1%), D87N (G259A, *n* = 7, 4.9%), D87Y (G259T, *n* = 6, 4.2%), and S83R (A247C and T249A, *n* = 4, 2.8%). Several other mutations occurring at lower frequencies, including D82N (G244A), D87H (G259C), and D87G (A260G), were each detected in one sample (*n* = 1, 0.7%). The WT sequence was found in nine samples (6.3%). In *gyrA*, the WT sequence was dominant, identified in 110 samples (77.5%). Mutations resulting in amino acid changes were observed less frequently in 32 samples (22.5%), with M95I (G285A/T) in 20 samples (14.1%), M95V (A283G) in 5 samples (3.5%), D99N (G295A) in 5 samples (3.5%), and G93C (G277T) in 2 samples (1.4%). Subgroup analyses were performed according to sex, age, risk of infection, and the presence or absence of symptoms (Table S2). The results showed that patients with a history of sexual activity with anonymous partners had a significantly higher frequency of A2058G mutant strains than those without such a history. In contrast, no significant differences were observed for the other factors analyzed.

**TABLE 2:** Frequency of mutations in drug resistance-associated genes in 142 samples^*d*^

Gene	DNA change	Amino acid change	No. (%)
23S rRNA	Mutation	NA	134 (94.3)
	A2058T	NA	11 (7.7)
	A2058G^*a*^	NA	29 (20.4)
	A2059G^*a*^	NA	94 (66.2)
	WT	NA	8 (5.6)
*parC*	Mutation	Mutation	133 (93.7)
	G244A	D82N	1 (0.7)
	A247C	S83R	2 (1.4)
	T249A	S83R	2 (1.4)
	G248A	S83N	20 (14.1)
	G248T	S83I	93 (65.5)
	G259A	D87N	7 (4.9)
	G259T	D87Y	6 (4.2)
	G259C	D87H	1 (0.7)
	A260G	D87G	1 (0.7)
	WT^*c*^	WT^*c*^	9 (6.3)
*gyrA*	Mutation	Mutation	32 (22.5)
	G277T^*b*^	G93C	2 (1.4)
	A283G	M95V	5 (3.5)
	G285A	M95I	16 (11.3)
	G285T	M95I	4 (2.8)
	G295A^*b*^	D99N	5 (3.5)
	WT	WT	110 (77.5)

^*a*^ Mixed infections of WT and drug resistance-associated mutations are indicated as (*n* = 3).

^*b*^ Mixed infections of WT and drug resistance-associated mutations are indicated as (*n* = 1).

^*c*^ WT also included a synonymous mutation, G244T (*n* = 1).

^*d*^ NA, not available; WT, wild-type.

### Analysis of dual-class drug resistance

Among the 142 samples, dual-class mutations in both 23S rRNA and *parC*, namely, MRMs and QRMs, respectively, were observed frequently (*n* = 127, 89.4%) (Table S3). Dual-QRMs, characterized by mutations in both *parC* and *gyrA*, were found in 32 samples (22.5%), with only two exceptions in which dual-class MRMs and QRMs were present. The most common genotype combination, namely, A2059G in 23S rRNA and S83I in *parC*, was found in 82 samples (57.7%), with 26 of these (18.3%) also carrying *gyrA* mutations. Although the overall proportion was small, mutations in *parC* (S83R and D87Y/N) were also associated with *gyrA* mutations. *mgpB-MG309* typing and phylogenetic analysis

Of the 160 samples that were sequenced for 23S rRNA, *parC*, and *gyrA*, 135 were fully typed at *mgpB-MG309*. Sequential samples from the same patient with identical STs were considered duplicates and excluded, resulting in 126 unique samples collected from 121 patients for analysis. Typing identified 27 distinct *mgpB* alleles, including 12 novel alleles (alleles 387–398), and 23 distinct *MG309* alleles, with 1 novel allele (allele 136). Combined *mgpB-MG309* typing revealed 61 distinct STs, including 41 novel STs (ST561, 581, 585, 588, 596, 600, 605–631, 633, and 635–640) (Table S4).

**Fig 2:**
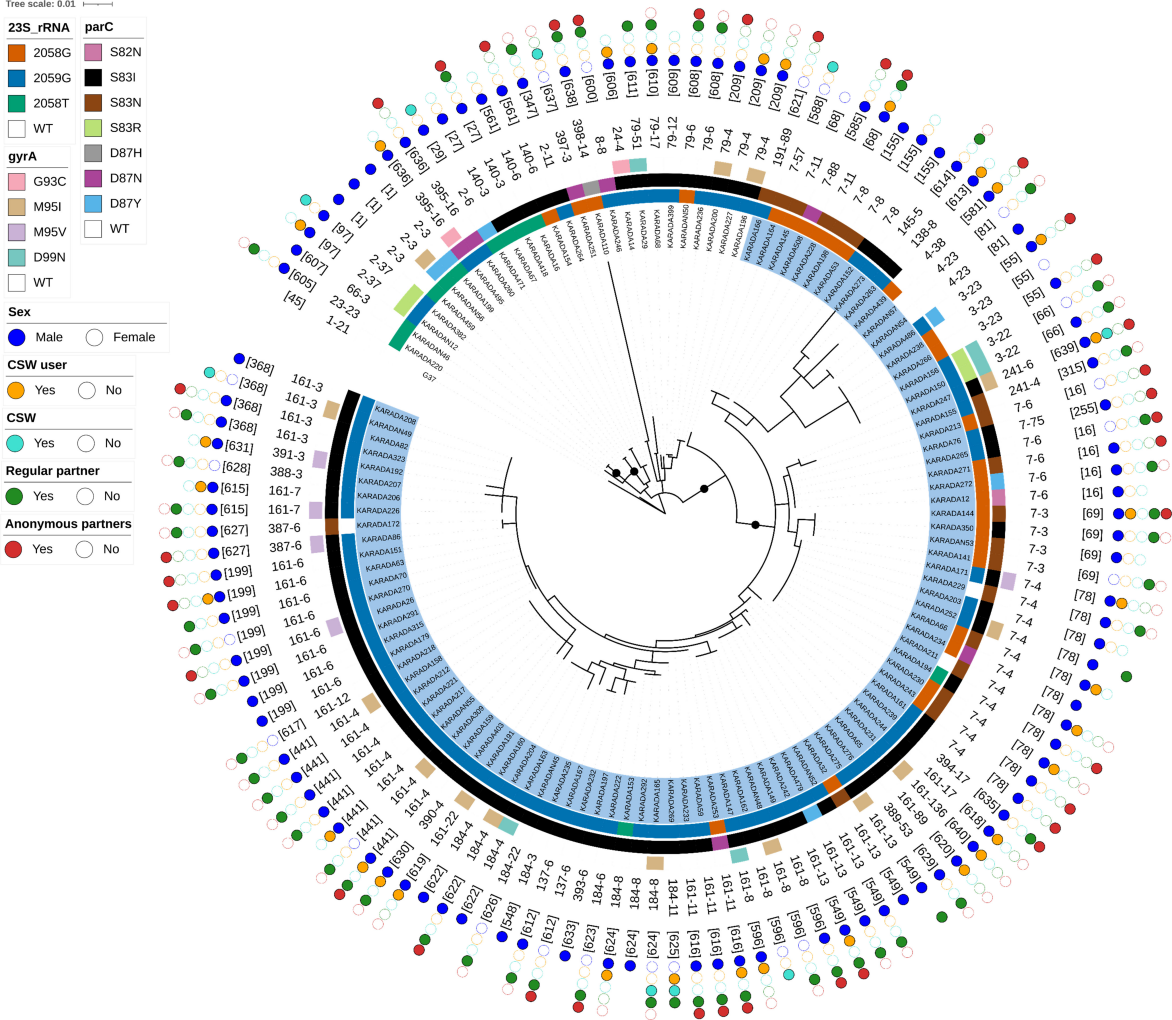
Phylogenetic tree of concatenated *mgpB* and *MG309* sequences. The phylogenetic tree was constructed using the maximum likelihood method based on 126 clinical samples. The G37 strain (GenBank: L43967.2) was used as a reference. Cluster B is highlighted in blue. From the inner to the outer rings, the plot displays the following information: 23S rRNA, *parC*, *gyrA*, and *mgpB–MG309* alleles, ST, sex, and risk of infection (including use of commercial sex workers [CSWs], working as a CSW, and sexual activity with regular or anonymous partners). Black circles on nodes represent >95% ultrafast bootstrap support. The scale bar indicates a branch length corresponding to a genetic distance of 0.01.

Phylogenetic analysis of concatenated *mgpB* and *MG309* sequences grouped the strains into two distinct clusters, A and B, with 95% ultrafast bootstrap support based on IQ-TREE analysis (Fig. 2). Cluster A (*n* = 27) was composed primarily of strains harboring *mgpB* alleles 2 (*n* = 7), 140 (*n* = 4), and 79 (*n* = 8). The most frequent STs within this cluster were ST1 (*mgpB-MG309* allele 2-3, *n* = 3) and ST209 (79-4, *n* = 3). All strains carrying *mgpB* alleles 140 and 79 exhibited dual-class resistance mutations of MRMs and *parC* S83I. The majority of strains analyzed in this study belonged to Cluster B (*n* = 99). The dominant *mgpB* alleles in this cluster were 7 (*n* = 28), 161 (*n* = 38), and 184 (*n* = 10). The most frequent ST was ST78 (7-4; *n* = 11). Except for three strains, all strains with *mgpB* alleles 161 and 184 harbored MRMs and the *parC* S83I mutation. Specifically, ST368 (161-3, *n* = 4), ST441 (161-4, *n* = 7), ST199 (161-6, *n* = 7), ST596 (161-8, *n* = 4), and ST622 (184-4, *n* = 3) were associated with a 100% prevalence of the 23S rRNA A2059G and *parC* S83I mutations. Among the novel STs, ST561, 596, 606, 608–620, 622–631, 633, 635, and 639–640 harbored MRM and *parC* S83I mutations in all samples, with the exception of a single ST627 sample (Table S4). Mutations in *gyrA* were detected sporadically across various *mgpB* alleles. No associations were observed between phylogenetic clustering and potential risk factors for infection.

### Association between QRMs and treatment outcome

We analyzed the relationship between QRMs and the outcome of quinolone-based therapy. Of the 128 patients who were treated with quinolone-based therapy, 39 were not evaluated with a TOC. Finally, we compared variables between the treatment failure group (*n* = 27) and treatment success group (*n* = 62) (Table 3). Except for five patients, sequential therapy with minocycline and sitafloxacin was administered. No differences were observed in terms of sex (*P* = 0.767) or age (*P* = 0.446). Regarding *parC* mutations, using WT as a reference, the S83I mutation was not associated with an increased treatment failure rate (37.3%, 22/59 vs 33.3%, 2/6, respectively, *P* = 1.000). For *gyrA* mutations, with WT as a reference, the mutant type was significantly associated with increased treatment failure (52.4%, 11/21 vs 23.5%, 16/68, respectively, *P* = 0.016). In the combinations of *parC* and *gyrA*, the treatment failure rate was higher in cases with *parC* S83I and *gyrA* mutations than in those with *parC* S83I and WT *gyrA*. With WT:WT as a reference, the S83I:mutant combination showed an increased treatment failure rate, but this was not statistically significant (50.0%, 10/20 vs 33.3%, 2/6, respectively, *P* = 0.652). There was no difference for the S83I:WT combination (30.8%, 12/39 vs 33.3%, 2/6, respectively, *P* = 1.000).

**TABLE 3:** Comparison of *parC* and *gyrA* mutations between the treatment failure and success cases^*a*^

Variable		Treatment outcome		*P*-value
		Failure (%)	Success (%)	
Sex, *n* (%)	Male	23/72 (31.9)	49/72 (68.1)	Reference
	Female	4/17 (23.5)	13/17 (76.5)	0.767
Age, years, median (interquartile range)		29 (24–35)	30 (27–35)	0.446
*parC, n* (%)				
	WT	2/6 (33.3)	4/6 (66.7)	Reference
	Mutant	25/83 (30.1)	58/83 (69.9)	1.000
	S83N	1/14 (7.1)	13/14 (92.9)	0.202
	D82N	0/–	1/1 (100)	–
	D87Y	0/–	4/4 (100)	–
	D87H	0/–	1/1 (100)	–
	S83R	1/3 (33.3)	2/3 (66.7)	–
	D87N	1/1 (100)	0/– (0)	–
	S83I	22/59 (37.3)	37/59 (62.7)	1.000
*gyrA, n* (%)				
	WT	16/68 (23.5)	52/68 (76.5)	Reference
	Mutant	11/21 (52.4)	10/21 (47.6)	0.016*
	M95I	6/15 (40.0)	9/15 (60.0)	0.208
	D99N	3/3 (100)	0/– (0)	–
	M95V	1/2 (50.0)	1/2 (50.0)	–
	G93C	1/1 (100)	0/– (0)	–
*parC:gyrA, n* (%)				
	WT:WT	2/6 (33.3)	4/6 (66.7)	Reference
	non-S83I mutant:WT	2/23 (8.7)	21/23 (91.3)	0.180
	non-S83I mutant:mutant	1/1 (100)	0/–	–
	S83I:WT	12/39 (30.8)	27/39 (69.2)	1.000
	S83I:mutant	10/20 (50.0)	10/20 (50.0)	0.652
Total		27/89 (30.3)	62/89 (69.7)	

^*a*^ WT, wild type. Asterisk indicates a significant difference. The Total row shows the overall number of cases with confirmed treatment outcomes (*n* = 89). For each variable, the denominators represent the total number of cases for that variable, while the numerators indicate the number of cases positive for that variable, categorized as either treatment failure or success. Percentages are calculated within each subgroup. “–” indicates that no cases were observed or data were not available for that subgroup.

## DISCUSSION

In this study, we demonstrated the high prevalence of MRMs and QRMs in MG collected in Tokyo, Japan, during the 2023–2025 period. Notably, MRMs were detected in 94.4% of the samples. The most common QRM was the *parC* S83I mutation, present in 65.5% of samples, while dual-QRMs, namely, mutations in *parC* and *gyrA*, were observed in 22.5% of cases. Phylogenetic analysis based on the *mgpB-MG309* region further revealed that strains harboring MRMs and *parC* S83I, indicative of dual-class resistance, clustered within specific *mgpB* alleles.

Deguchi et al. analyzed 627 clinical samples collected between 2013 and 2017 in Japan and reported a marked rise in resistance-associated mutations during this period. Specifically, the prevalence of MRMs increased from approximately 40% to 70%, while the *parC* S83I mutation rate rose from 20% to 30%, and *gyrA* mutations increased from 2% to 10% (12). More recently, Ando et al. examined 180 clinical samples collected from MSM between 2019 and 2022 in Tokyo and found that the prevalence of resistance-associated mutations continued to rise. Their study reported an MRM prevalence of 89.4%, *parC* S83I at 71.4%, and *gyrA* mutations at 22.5% (10). Our findings closely align with those of Ando et al., indicating that resistance rates have continued to increase in the 2020s compared with the 2010s. Although our study did not collect information on MSM status, we observed a high rate of drug-resistant MG in our general population cohort of men and women, suggesting that the spread of drug-resistant MG is not confined to MSM.

Previous reports on MG strain diversity in Japan during the 2010s are limited. Kikuchi et al. analyzed 20 strains collected from men with urethritis in Sendai between 2011 and 2012, reporting MRMs in 25.0% of strains and *parC* S83I mutations in 15.0% (20). In that study, *mgpB* allele 7 (40.0%) was the most frequent, corresponding to Cluster B, followed by alleles 21 (15.0%, Cluster A) and 14 (15.0%, Cluster B). In contrast, although allele 7 remained commonly detected in the present study, there was also a high prevalence of *mgpB* alleles 161, 184, 79, and 140, which harbored a high frequency of dual-class resistance mutations, including MRMs and *parC* S83I, indicating a shift in the predominant circulating strains over the past decade. This shift may reflect the expansion of multiple clonal complexes of dual-class drug-resistant strains in Japan, potentially driven by antibiotic selective pressure. A similar phenomenon has been described in France, where Pereyre et al. (26) reported the emergence of *mgpB* type ST159 (closest to allele 140 in this study), which harbored dual-class drug-resistant 23S rRNA 2059T and *parC* S83I in 2021–2022. Unlike *parC* S83I, *gyrA* mutations were found sporadically and were not cluster-specific, suggesting that dual-QRMs may be emerging independently across different genetic backgrounds. These findings raise concerns about the spread of genetically diverse, dual-class drug-resistant clonal complexes, emphasizing the need for cross-regional molecular surveillance.

In the treatment of MG, sequential therapy using tetracycline-macrolide or tetracycline-fluoroquinolone combinations is gaining attention (27). The cure rate for MG with doxycycline monotherapy is 30%–40% (28), but doxycycline treatment has been shown to reduce the bacterial load of MG (27). In Melbourne, Australia, during 2016–2017, a treatment strategy involving doxycycline pretreatment followed by the administration of azithromycin or sitafloxacin/moxifloxacin based on MRMs achieved a cure rate of over 92% (27). This approach was applied to a population in which two-thirds of cases harbored MRMs, and 20% of those with MRMs also had QRMs (8, 9, 27). In contrast, the overall cure rate in the present study was lower, at 69.7% (62 out of 89 patients), than in the Australian study. The high prevalence of *parC* S83I and *gyrA* mutations in our study population is likely to have contributed to the lower cure rate. Several studies have demonstrated that the *parC* S83I mutation is associated with an increased risk of quinolone treatment failure, with failure rates ranging from 21.1% to 60% (7, 10, 23). Furthermore, when *parC* S83I and *gyrA* mutations are present, the treatment failure rate rises even further, reaching 58.3% to 81.2% (7, 10, 23). Our findings are consistent with previous studies, in which the treatment failure rate was 37.3% for the *parC* S83I mutation overall but rose to 50% in the presence of a *gyrA* mutation. However, the TOC implementation rate in our study was 42.0%, likely reflecting the lack of clear national recommendations, patient non-attendance after symptom improvement, and testing at other facilities. This limitation may have led to an overestimation of treatment failure and mutation impact. Further studies are needed to clarify the relationship between *parC* S83I and *gyrA* mutations and treatment outcome.

With the increasing rate of treatment failure, refractory cases that do not respond to multiple courses of antibiotic therapy have been on the rise. In our study, up to seven courses of treatment were required for cure in some refractory cases. The occurrence of refractory infections may have been influenced not only by the presence of drug resistance genes but also by factors such as medication adherence and reinfection. In addition, the lack of the full standardization of initial and retreatment regimens, with antibiotic selection largely dependent on the discretion of individual physicians, may have contributed to the occurrence of refractory cases. A third-line treatment regimen has yet to be established; however, several options for refractory cases have been recommended, including pristinamycin and long-term minocycline and doxycycline (7, 28). In this study, the majority of patients were cured through the re-administration of sequential therapy with minocycline and sitafloxacin and long-term minocycline. Therefore, these regimens may be valuable options when initial therapy fails. The exploration of effective third-line treatments should be a priority.

The major limitation of this study is that sample collection was conducted only at clinics in Tokyo, Japan. Given that previous studies have identified geographical differences in antimicrobial resistance rates even in the same country (12), our findings may not fully reflect the nationwide distribution of circulating genotypes in Japan. Additionally, data on whether participants were MSM were not available in our study. Therefore, we were unable to determine whether differences in drug resistance or treatment failure rates are associated with MSM status. Further epidemiological studies involving a broader range of regions are warranted to understand better the national trends in resistance and strain distribution. Further nationwide epidemiological studies are also needed to clarify resistance and strain distribution. Integrating 23S rRNA, *parC*, and *gyrA* sequencing and *mgpB–MG309* typing into routine surveillance would enable the early detection of resistant strains, tracking of transmission clusters, and optimization of empirical treatment when resistance-guided therapy is unavailable.

In conclusion, we found the high prevalence of drug resistance-associated mutations in MG in Tokyo, Japan. Notably, potential dual-class drug-resistant clonal complexes with MRMs and the *parC* S83I mutation, particularly those harboring *mgpB* alleles 79, 140, 161, and 184, appear to be emerging. In settings with a high prevalence of MRMs, azithromycin should be avoided as a first-line treatment, and fluoroquinolone-based therapy may be preferred. When *parC* S83I and *gyrA* mutations are prevalent, fluoroquinolone therapy can still be effective, but the risk of treatment failure should be considered. In cases of treatment failure, long-term tetracycline therapy or the reintroduction of fluoroquinolone-based therapy may be appropriate. To maintain treatment success rates, it is essential to promote the appropriate use of antibiotics and implement ongoing surveillance to prevent a further increase in drug resistance-associated mutations in MG.

## Data Availability

The datasets analyzed during the present study are available from the corresponding author on reasonable request.

## SUPPLEMENTAL MATERIAL

Table S1 aac.01367-25-s0001.pdf Primers used in this study.

Table S2 aac.01367-25-s0002.xlsx Subgroup analysis of the frequency of drug resistance-associated mutations in 142 clinical samples.

Table S3 aac.01367-25-s0003.pdf Frequency of dual-class drug resistance-associated mutations in 142 clinical samples.

Table S4 aac.01367-25-s0004.xlsx Results of *mgpB*-*MG309* typing in this study.

ASM does not own the copyrights to Supplemental Material that may be linked to, or accessed through, an article. The authors have granted ASM a non-exclusive, world-wide license to publish the Supplemental Material files. Please contact the corresponding author directly for reuse.
