# Supplementary material for: Spread of dual-class drug-resistant Mycoplasma genitalium in Tokyo, Japan, 2023–2025
Source: Antimicrob Agents Chemother. 2025 Dec 30;70(2):e01367-25. doi: 10.1128/aac.01367-25 (PMC12888857; doi:10.1128/aac.01367-25)
Supplement: Table S1 — Primers used in this study. [file aac.01367-25-s0001.pdf]

Supplementary Table S1. Primers used in this study.

Primer name	Target gene	Sequence (5′–3′)	Application in this study	Reference
23SrRNA_1st_F	23S rRNA	GGAGGTTAGCAATTTATTGCAAA	Nested PCR	[1]
23SrRNA_1st_R	23S rRNA	CCACCTAACACTGTCTTGAAACTGG	Nested PCR	[1]
23SrRNA_2nd_F	23S rRNA	CGTAACTATAACGGTCCTAAGGTAG	Nested PCR and sequencing	[1]
23SrRNA_2nd_R	23S rRNA	CACATCAACAAATCCTTGCGAAC	Nested PCR and sequencing	[1]
*parC*_1st_F	*parC*	AAACCAGTACAAAGACGGATCT	Nested PCR	[2]
*parC*_1st_R	*parC*	GAGGTTAGGCAGTAAGGTTGG	Nested PCR	[2]
*parC*_2nd_F	*parC*	TGGGCTTAAAACCCACCACT	Nested PCR and sequencing	[3]
*parC*_2nd_R	*parC*	CGGGTTTCTGTGTAACGCAT	Nested PCR and sequencing	[3]
*gyrA*_1st_F	*gyrA*	TGTTGCTCGTGCTTTACCTGA	Nested PCR	[2]
*gyrA*_1st_R	*gyrA*	AAGCTGCTGGTAGAACAGTTGG	Nested PCR	[2]
*gyrA*_2nd_F	*gyrA*	CGTCGTGTTCTTTATGGTGC	Nested PCR and sequencing	[3]
*gyrA*_2nd_R	*gyrA*	ATAACGYYGTGCAGCAGGTC	Nested PCR and sequencing	[3]
MgPa-1	*MgpB*	AGTTGATGAAACCTTAACCCCTTGG	Nested PCR	[4]
MgPa-3	*MgpB*	CCGTTGAGGGGTTTTCCATTTTTGC	Nested PCR	[4]
MgPa-2nd-F	*MgpB*	TGATGAAACCTTAACCCCTTGGACT	Nested PCR and sequencing	This study
MgPa-2nd-R	*MgpB*	TTGAGGGGTTTTCCATTTTTGCTAA	Nested PCR and sequencing	This study
384275F	*MG309*	GTGCTAGAGAAGTGTTTCTAGGATC	Semi-nested PCR	[5]
384655R	*MG309*	AACTAGCAGAACGTAACCAACC	Semi-nested PCR and sequencing	[5]
384330F	*MG309*	TTGTGAATATTGCGGTGAGG	Semi-nested PCR and sequencing	[5]

References

[1]. Getman D, Jiang A, O'Donnell M, Cohen S. 2016. *Mycoplasma genitalium* Prevalence, Coinfection, and Macrolide Antibiotic Resistance Frequency in a Multicenter Clinical Study Cohort in the United States. *J Clin Microbiol* 54:2278-83.

[2]. Murray GL, Bodiyabadu K, Danielewski J, Garland SM, Machalek DA, Fairley CK, Jensen JS, Williamson DA, Tan LY, Mokany E, Durukan D, Bradshaw CS. 2020. Moxifloxacin and Sitafloxacin Treatment Failure in *Mycoplasma genitalium* Infection: Association with *parC* Mutation G248T (S83I) and Concurrent *gyrA* Mutations. *J Infect Dis* 221:1017-1024.
[3]. Shimada Y, Deguchi T, Nakane K, Masue T, Yasuda M, Yokoi S, Ito S, Nakano M, Ito S, Ishiko H. 2010. Emergence of Clinical Strains of *Mycoplasma genitalium* Harbouring Alterations in ParC Associated with Fluoroquinolone Resistance. *Int J Antimicrob Agents* 36:255-8.
[4]. Jensen JS, Uldum SA, Søndergård-Andersen J, Vuust J, Lind K. 1991. Polymerase Chain Reaction for Detection of *Mycoplasma genitalium* in Clinical Samples. *J. Clin. Microbiol* 29:46-50.
[5]. Ma L, Martin DH. 2004. Single-Nucleotide Polymorphisms in the rRNA Operon and Variable Numbers of Tandem Repeats in the Lipoprotein Gene among *Mycoplasma genitalium* Strains from Clinical Specimens. *J Clin Microbiol* 42:4876-4878.
