# Supplementary material for: Spread of dual-class drug-resistant Mycoplasma genitalium in Tokyo, Japan, 2023–2025
Source: Antimicrob Agents Chemother. 2025 Dec 30;70(2):e01367-25. doi: 10.1128/aac.01367-25 (PMC12888857; doi:10.1128/aac.01367-25)
Supplement: Table S3 — Frequency of dual-class drug resistance-associated mutations in 142 clinical samples. [file aac.01367-25-s0003.pdf]

Supplementary Table S3. Frequency of dual-class drug resistance-associated mutations in 142 clinical samples.

DNA/amino acid change			Drug resistance profile, no. (%)					
							Dual-class	
**23S rRNA**	***parC***	***gyrA***	**Total**	**MRMs**	**QRMs**	**Dual-QRMs**	**MRMs +QRMs**	**MRMs +dual-QRMs**
WT	WT	WT	2 (1.4)	–	–	–	–	–
WT	S83I	M95I	2 (1.4)	–	2 (1.4)	2 (1.4)	–	–
WT	D87G/N	WT	4 (2.8)	–	4 (2.8)	–	–	–
A2058G	WT	WT	3 (2.1)	3 (2.1)	–	–	–	–
A2058G	S83I	WT	4 (2.8)	4 (2.8)	4 (2.8)	–	4 (2.8)	–
A2058G	D82N<br>S83N<br>D87H/N/Y	WT	22 (15.5)	22 (15.5)	22 (15.5)	–	22 (15.5)	–
A2058T	WT	WT	3 (2.1)	3 (2.1)	–	–	–	–
A2058T	S83I	WT	5 (3.5)	5 (3.5)	5 (3.5)	–	5 (3.5)	–
A2058T	D87Y	WT	2 (1.4)	2 (1.4)	2 (1.4)	–	2 (1.4)	–
A2058T	D87Y	M95I	1 (0.7)	1 (0.7)	1 (0.7)	1 (0.7)	1 (0.7)	1 (0.7)
A2059G	WT	WT	1 (0.7)	1 (0.7)	–	–	–	–
A2059G	S83I	WT	56 (39.4)	56 (39.4)	56 (39.4)	–	56 (39.4)	–
A2059G	S83I	G93C<br>M95I/V<br>D99N	26 (18.3)	26 (18.3)	26 (18.3)	26 (18.3)	26 (18.3)	26 (18.3)
A2059G	S83N/R<br>D87N/Y	WT	8 (5.6)	8 (5.6)	8 (5.6)	–	8 (5.6)	–
A2059G	S83R<br>D87N	G93C<br>D99N	3 (2.1)	3 (2.1)	3 (2.1)	3 (2.1)	3 (2.1)	3 (2.1)
Total	-	-	142 (100)	134 (94.4)	133 (93.7)	32 (22.5)	127 (89.4)	30 (21.1)

MRMs, macrolide resistance-associated mutations; QRMs, fluoroquinolone resistance-associated mutations; WT, wild-type.
